# Introduction of a Polyethylene Glycol Linker Improves Uptake of ^67^Cu-NOTA-Conjugated Lactam-Cyclized Alpha-Melanocyte-Stimulating Hormone Peptide in Melanoma

**DOI:** 10.3390/cancers15102755

**Published:** 2023-05-14

**Authors:** Zheng Qiao, Jingli Xu, Darrell R. Fisher, Rene Gonzalez, Yubin Miao

**Affiliations:** 1 Department of Radiology, University of Colorado Denver, Aurora, CO 80045, USA; 2 Versant Medical Physics and Radiation Safety, Richland, WA 99354, USA; 3 Department of Medical Oncology, University of Colorado Denver, Aurora, CO 80045, USA

**Keywords:** ^67^Cu-NOTA, lactam-cyclized, alpha-melanocyte-stimulating hormone, melanocortin-1 receptor, melanoma treatment

## Abstract

**Simple Summary:**

There is a need to develop new theranostic approaches for malignant melanoma. Only 35% of patients with metastatic melanoma reach the milestone of 5-year survival, despite the success of new immunotherapy. We have developed a new class of peptides to target melanocortin-1 receptors (MC1Rs) that display elevated levels in human melanoma. In this study, we examined the melanoma targeting and biodistribution properties of two ^67^Cu-tagged peptides in tumor-bearing mice. We found that one of the peptides, namely ^67^Cu-NOTA-PEG_2_Nle-CycMSH_hex_, exhibited favorable melanoma targeting and biodistribution properties that underscored its potential as an MC1R-targeted therapeutic peptide for melanoma treatment in the future.

**Abstract:**

The aim of this study was to evaluate the effect of linker on tumor targeting and biodistribution of ^67^Cu-NOTA-PEG_2_Nle-CycMSH_hex_ {^67^Cu-1,4,7-triazacyclononane-1,4,7-triyl-triacetic acid-polyethylene glycol-Nle-c[Asp-His-DPhe-Arg-Trp-Lys]-CONH_2_} and ^67^Cu-NOTA-GGNle-CycMSH_hex_ {^67^Cu-NOTA-GlyGlyNle-CycMSH_hex_} on melanoma-bearing mice. NOTA-PEG_2_Nle-CycMSH_hex_ and NOTA-GGNle-CycMSH_hex_ were synthesized and purified by HPLC. The biodistribution of ^67^Cu-NOTA-PEG_2_Nle-CycMSH_hex_ and ^67^Cu-NOTA-GGNle-CycMSH_hex_ was determined in B16/F10 melanoma-bearing C57 mice. The melanoma imaging property of ^67^Cu-NOTA-PEG_2_Nle-CycMSH_hex_ was further examined in B16/F10 melanoma-bearing C57 mice. ^67^Cu-NOTA-PEG_2_Nle-CycMSH_hex_ exhibited higher tumor uptake than ^67^Cu-NOTA-GGNle-CycMSH_hex_ at 2, 4, and 24 h post-injection. The tumor uptake of ^67^Cu-NOTA-PEG_2_Nle-CycMSH_hex_ was 27.97 ± 1.98, 24.10 ± 1.83, and 9.13 ± 1.66% ID/g at 2, 4, and 24 h post-injection, respectively. Normal organ uptake of ^67^Cu-NOTA-PEG_2_Nle-CycMSH_hex_ was lower than 2.6% ID/g at 4 h post-injection, except for kidney uptake. The renal uptake of ^67^Cu-NOTA-PEG_2_Nle-CycMSH_hex_ was 6.43 ± 1.31, 2.60 ± 0.79, and 0.90 ± 0.18% ID/g at 2, 4, and 24 h post-injection, respectively. ^67^Cu-NOTA-PEG_2_Nle-CycMSH_hex_ showed high tumor to normal organ uptake ratios after 2 h post-injection. The B16/F10 melanoma lesions could be clearly visualized by single photon emission computed tomography (SPECT) using ^67^Cu-NOTA-PEG_2_Nle-CycMSH_hex_ as an imaging probe at 4 h post-injection. The favorable tumor targeting and biodistribution properties of ^67^Cu-NOTA-PEG_2_Nle-CycMSH_hex_ underscored its potential as an MC1R-targeted therapeutic peptide for melanoma treatment.

## 1. Introduction

Malignant melanoma continues to be the most deadly form of skin cancer, with approximately 97,610 new cases and 7990 deaths in the United States in 2023 [1]. Molecularly-targeted melanoma therapies, including BRAF inhibitors (Vemurafenib), cytotoxic T-lymphocyte antigen 4 (CTLA-4) inhibitors (Ipilimumab), and programmed death-1 receptor (PD-1) inhibitors (Nivolumab), have significantly extended the overall survival of metastatic melanoma patients by months over the past decade [2,3,4,5,6,7]. However, the 5-year survival of metastatic melanoma patients remains at only 35% despite the successes of the aforementioned new treatments [7]. There is a need to develop new theranostic approaches for malignant melanoma.

Melanocortin-1 receptor (MC1R) is a G protein-coupled receptor that expresses at elevated levels in human melanotic and amelanotic melanoma [8,9,10]. Meanwhile, α-melanocyte-stimulating hormone (α-MSH) peptides can bind to MC1Rs with nanomolar binding affinities [11,12,13,14,15,16,17,18,19,20]. Therefore, MC1R is an attractive molecular target for developing MC1R-targeted theranostic α-MSH peptides. Over the past years, we have developed a new class of MC1R-targeted peptide radiopharmaceuticals that include the core structure of Gly-Gly-Nle-c[Asp-His-DPhe-Arg-Trp-Lys]-CONH_2_ (GGNle-CycMSH_hex_) for melanoma imaging and therapy [11,12,13,14,15,16,17,18,19,20]. Our first-in-human melanoma imaging work using ^68^Ga-DOTA-GGNle-CycMSH_hex_ clearly detected human metastatic melanoma lesions in the brain, lung, connective tissue, and intestines [10], demonstrating the clinical relevance of MC1R for melanoma imaging, as well as for potential MC1R-targeted radionuclide therapy.

Due to the emissions of positrons and beta-particles, ^64^Cu (T_1/2_ = 12.7 h, 17.4% *β*^+^, 40% *β*^−^) is an attractive theranostic radionuclide. NOTA is a better chelator for ^64^Cu than DOTA because of the superior in vivo stability of ^64^Cu-NOTA compared to ^64^Cu-DOTA. In our previous work, ^64^Cu-NOTA-GGNle-CycMSH_hex_ {^64^Cu-1,4,7-triazacyclononane-1,4,7-triacetic acid-GGNle-CycMSH_hex_) showed higher B16/F1 melanoma uptake by 2.4 times and less liver uptake by 93% than ^64^Cu-DOTA-GGNle-CycMSH_hex_ at 2 h post-injection [14]. Meanwhile, we reported that the substitution of the GG linker with an Aoc or polyethylene glycol (PEG) linker could dramatically affect the melanoma uptake of ^64^Cu-NOTA-AocNle-CycMSH_hex_ and ^64^Cu-NOTA-PEG_2_Nle-CycMSH_hex_ peptides [21]. Interestingly, the B16/F10 melanoma uptake of ^64^Cu-NOTA-PEG_2_Nle-CycMSH_hex_ was 2.5 times the tumor uptake of ^64^Cu-NOTA-AocNle-CycMSH_hex_ at 2 h post-injection [21].

Recently, ^67^Cu (T_1/2_ = 61.8 h, 100% *β*^−^, E_max_ = 562 keV, E_mean_ = 141 keV) has obtained renewed interest as an attractive therapeutic radionuclide [22,23,24,25,26,27,28]. ^67^Cu and ^64^Cu are matched-pair theranostic radionuclides that share identical coordination chemistry. Moreover, ^67^Cu emits γ-photons (93 keV, 16% and 185 keV, 49%) that are suitable for SPECT imaging. Therefore, the follow-up ^67^Cu SPECT imaging provides the opportunity for dosimetry calculation after treatment. In this study, we prepared ^67^Cu-NOTA-PEG_2_Nle-CycMSH_hex_ and ^67^Cu-NOTA-GGNle-CycMSH_hex_ peptides and determined their melanoma targeting and biodistribution properties in B16/F10 melanoma-bearing C57 mice. Since ^67^Cu-NOTA-PEG_2_Nle-CycMSH_hex_ displayed higher tumor uptake than ^67^Cu-NOTA-GGNle-CycMSH_hex_ after 2 h post-injection, we further examined the melanoma imaging of ^67^Cu-NOTA-PEG_2_Nle-CycMSH_hex_ in B16/F10 melanoma-bearing C57 mice.

## 2. Materials and Methods

### 2.1. Chemicals and Reagents

Amino acids and resin were purchased from Advanced ChemTec Inc. (Louisville, KY, USA) and Novabiochem (San Diego, CA, USA). NOTA(OtBu)_2_ was purchased from the CheMatech Inc. (Dijon, France) for peptide synthesis. ^67^CuCl_2_ was purchased from Idaho Accelerator Center at Idaho State University (Pocatello, ID, USA) for radiolabeling. B16/F10 murine melanoma cells were received from the American Type Culture Collection (Manassas, VA, USA). All other chemicals used in this study were purchased from Thermo Fisher Scientific (Waltham, MA, USA) and used without further purification.

### 2.2. Peptide Synthesis and Radiolabeling

NOTA-PEG_2_Nle-CycMSH_hex_ and NOTA-GGNle-CycMSH_hex_ were synthesized and characterized by liquid chromatography-mass spectrometry (LC-MS) according to our previous publication [21]. Generally, 210 µmol of each fluorenylmethoxycarbonyl (Fmoc)-protected amino acid and NOTA(OtBu)_2_ and 70 µmol of resin were used for the synthesis. ^67^Cu-NOTA-PEG_2_Nle-CycMSH_hex_ and ^67^Cu-NOTA-GGNle-CycMSH_hex_ were prepared using 0.5 M NH_4_OAc (pH 5.4). Briefly, 10 μL of ^67^CuCl_2_ (37–74 MBq in 0.01 M HCl aqueous solution), 10 μL of 1 mg/mL peptide aqueous solution, and 200 μL of 0.5 M NH_4_OAc (pH 5.4) were added into a reaction vial and incubated at 75 °C for 1 h. Thereafter, 10 μL of 0.5% EDTA aqueous solution was added to scavenge potentially unbound ^67^Cu^2+^. ^67^Cu-NOTA-PEG_2_Nle-CycMSH_hex_ and ^67^Cu-NOTA-GGNle-CycMSH_hex_ complexes were purified by Waters RP-HPLC (Milford, MA, USA) on a Grace Vydac C-18 reverse-phase analytical column (Deerfield, IL, USA) with a flow rate of 1 mL/min. A 20 min gradient of 20–30% acetonitrile in a 20 mM HCl aqueous solution was used for peptide purification. Each collected peptide solution was purged with N_2_ gas for 15 min to remove the acetonitrile, then adjusted to pH 7.4 with 0.1 M NaOH and sterile saline for cellular binding and animal studies.

### 2.3. Specific Binding

Specific binding of ^67^Cu-NOTA-PEG_2_Nle-CycMSH_hex_ and ^67^Cu-NOTA-GGNle-CycMSH_hex_ was determined on B16/F10 melanoma cells according to our previous publication [21]. Briefly, the cells were incubated at 25 °C for 1 h with approximately 0.01 MBq of ^67^Cu-NOTA-PEG_2_Nle-CycMSH_hex_ and ^67^Cu-NOTA-GGNle-CycMSH_hex_ with or without 10 μg (6.07 nmol) of unlabeled [Nle^4^, D-Phe^7^]-α-MSH (NDP-MSH). The binding medium was aspirated after incubation. The cells were washed twice with 0.5 mL of ice-cold 0.01 M phosphate-buffered saline (PBS) buffer containing 0.2% BSA (pH = 7.4) and collected and measured in a Wallac 1480 automated gamma counter (PerkinElmer, NJ, USA).

### 2.4. Biodistribution and Imaging Studies

All animal studies were conducted in compliance with Institutional Animal Care and Use Committee approval. C57 mice were purchased from Charles River Laboratory (Wilmington, NC). B16/F10 melanoma-bearing C57 mice were generated according to our previous publication [21]. Each melanoma-bearing mouse was injected with 0.037 MBq of ^67^Cu-NOTA-PEG_2_Nle-CycMSH_hex_ or ^67^Cu-NOTA-GGNle-CycMSH_hex_ via the tail vein. The tumor uptake specificity of ^67^Cu-NOTA-PEG_2_Nle-CycMSH_hex_ and ^67^Cu-NOTA-GGNle-CycMSH_hex_ was determined by co-injecting 10 μg (6.07 nmol) of unlabeled NDP-MSH. Mice were sacrificed at 0.5, 2, 4, and 24 h post-injection, and tumors and organs of interest were harvested, weighed, and counted. The blood value was taken as 6.5% of the whole-body weight.

The melanoma imaging property of ^67^Cu-NOTA-PEG_2_Nle-CycMSH_hex_ was further examined in B16/F10 melanoma-bearing C57 mice. Each mouse was injected with 7.4 MBq of ^67^Cu-NOTA-PEG_2_Nle-CycMSH_hex_ via the tail vein. Imaging studies were performed 4 h post-injection. Reconstructed SPECT data was visualized using VivoQuant (Invicro, Boston, MA, USA).

### 2.5. Statistical Analysis

Statistical analysis was performed using the Microsoft Office Excel 2007 Student’s *t* test for unpaired data. A 95% confidence level was chosen to determine the significance of differences in tumor and kidney uptake between ^67^Cu-NOTA-PEG_2_Nle-CycMSHhex with/without NDP-MSH blockade and tumor and kidney uptake between ^67^Cu-NOTA-GGNle-CycMSH_hex_ with/without NDP-MSH blockade. The differences at the 95% confidence level (*p* < 0.05) were considered significant.

## 3. Results

**Figure 1:**
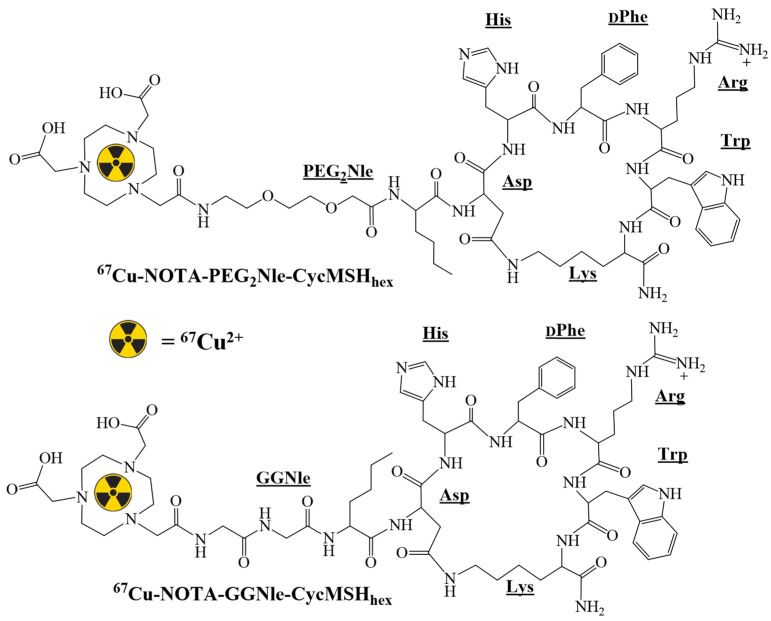
Schematic structures of ^67^Cu-NOTA-PEG_2_Nle-CycMSH_hex_ and ^67^Cu-NOTA-GGNle-CycMSH_hex_.

**Figure 2:**
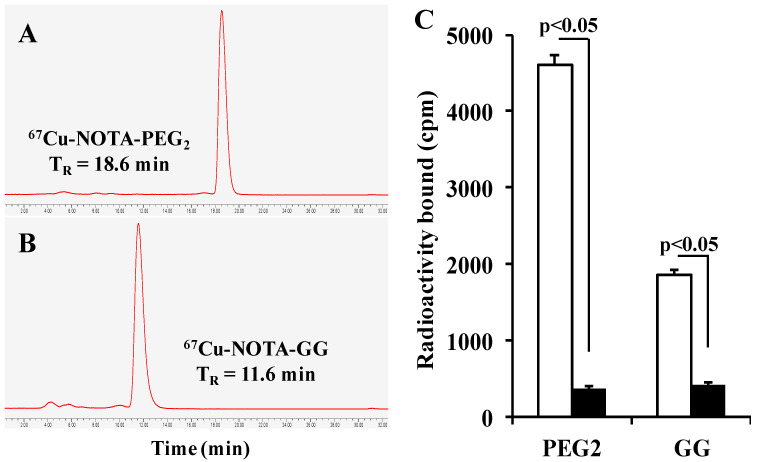
Radioactive HPLC profiles of ^67^Cu-NOTA-PEG_2_Nle-CycMSH_hex_ (**A**) and ^67^Cu-NOTA-GGNle-CycMSH_hex_ (**B**). The retention times of ^67^Cu-NOTA-PEG_2_Nle-CycMSH_hex_ and ^67^Cu-NOTA-GGNle-CycMSH_hex_ were 18.6 and 11.6 min, respectively. Specific binding (**C**) of ^67^Cu-NOTA-PEG_2_Nle-CycMSH_hex_ and ^67^Cu-NOTA-GGNle-CycMSH_hex_ on B16/F10 melanoma cells with (black) and without (white) peptide blockade, respectively.

The schematic structures of NOTA-PEG_2_Nle-CycMSH_hex_ and NOTA-GGNle-CycMSH_hex_ are presented in Figure 1. Both peptides were synthesized, purified by HPLC, and displayed greater than 90% purities after HPLC purification. ^67^Cu-NOTA-PEG_2_Nle-CycMSH_hex_ and ^67^Cu-NOTA-GGNle-CycMSH_hex_ were prepared in a 0.5 M NH_4_OAc-buffered solution with greater than 90% radiochemical yields. The radioactive HPLC profiles of ^67^Cu-NOTA-PEG_2_Nle-CycMSH_hex_ and ^67^Cu-NOTA-GGNle-CycMSH_hex_ are shown in Figure 2. The retention times of ^67^Cu-NOTA-PEG_2_Nle-CycMSH_hex_ and ^67^Cu-NOTA-GGNle-CycMSH_hex_ were 18.6 and 11.6 min, respectively. The specific activity was 560 mCi/µg for ^67^Cu-NOTA-PEG_2_Nle-CycMSH_hex_ and ^67^Cu-NOTA-GGNle-CycMSH_hex_. As shown in Figure 2B, both ^67^Cu-NOTA-PEG_2_Nle-CycMSH_hex_ and ^67^Cu-NOTA-GGNle-CycMSH_hex_ exhibited MC1R-specific binding. The peptide blockade reduced 92% of ^67^Cu-NOTA-PEG_2_Nle-CycMSH_hex_ and 72% of ^67^Cu-NOTA-GGNle-CycMSH_hex_ cellular uptake.

Table 1 and Table 2 show the biodistribution results of ^67^Cu-NOTA-PEG_2_Nle-CycMSH_hex_ and ^67^Cu-NOTA-GGNle-CycMSH_hex_. ^67^Cu-NOTA-PEG_2_Nle-CycMSH_hex_ displayed rapid melanoma uptake and prolonged tumor retention. The tumor uptake of ^67^Cu-NOTA-PEG_2_Nle-CycMSH_hex_ was 8.83 ± 2.19, 27.97 ± 1.98, 24.10 ± 1.83, and 9.13 ± 1.66% ID/g at 0.5, 2, 4, and 24 h post-injection, respectively. Approximately 94% of tumor uptake of ^67^Cu-NOTA-PEG_2_Nle-CycMSH_hex_ was blocked by 10 μg (6.07 nmol) of NDP-MSH (*p* < 0.05), suggesting that the tumor uptake was MC1R-mediated. Normal organ uptake of ^67^Cu-NOTA-PEG_2_Nle-CycMSH_hex_ was lower than 2% ID/g at 2 h post-injection, except for kidney uptake (6.34 ± 1.63% ID/g). The renal uptake of ^67^Cu-NOTA-PEG_2_Nle-CycMSH_hex_ decreased to 2.60 ± 0.79 and 0.90 ± 0.18% ID/g at 4 and 24 h post-injection.

^67^Cu-NOTA-GGNle-CycMSH_hex_ exhibited lower tumor uptake than ^67^Cu-NOTA-PEG_2_Nle-CycMSH_hex_ at 2, 4, and 24 h post-injection. The tumor uptake of ^67^Cu-NOTA-GGNle-CycMSH_hex_ was 16.58 ± 1.40, 11.66 ± 1.94, 8.79 ± 1.31, and 4.92 ± 1.58% ID/g at 0.5, 2, 4, and 24 h post-injection, respectively. Approximately 95% of tumor uptake of ^67^Cu-NOTA-GGNle-CycMSH_hex_ was decreased by 10 μg (6.07 nmol) of NDP-MSH blockade (*p* < 0.05), indicating that the tumor uptake was MC1R-specific. Normal organ uptake of ^67^Cu-NOTA-GGNle-CycMSH_hex_ was lower than 2% ID/g at 2 h post-injection, except for kidney uptake (5.08 ± 1.52% ID/g). The renal uptake of ^67^Cu-NOTA-GGNle-CycMSH_hex_ was 4.21 ± 0.92 and 1.19 ± 0.28% ID/g at 4 and 24 h post-injection.

**Figure 3:**
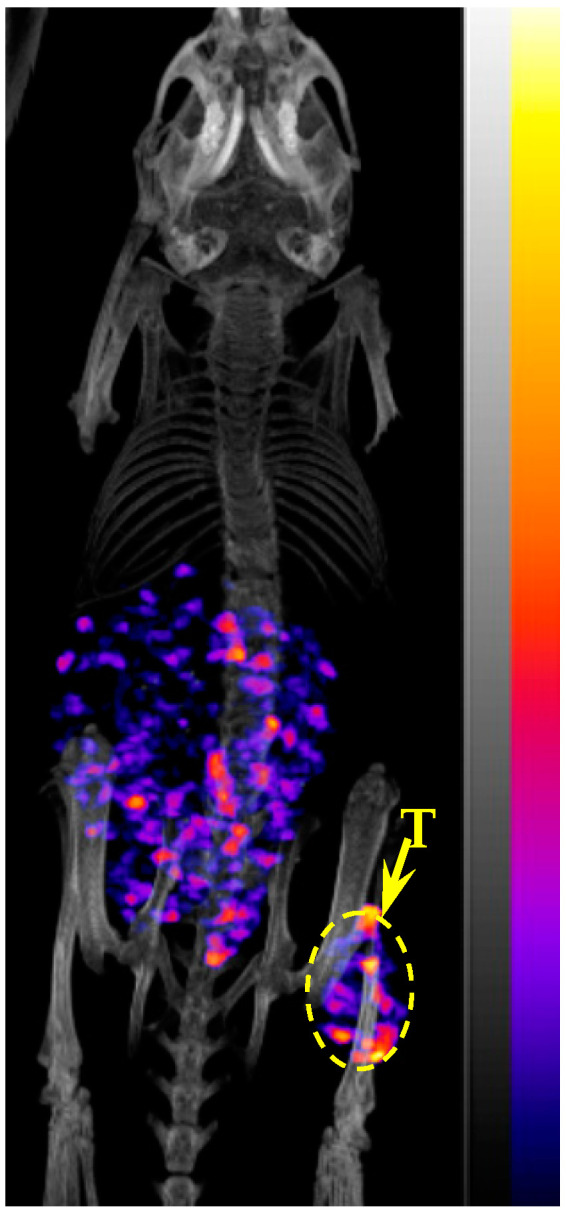
Representative maximum intensity projection SPECT/CT image of a B16/F10 melanoma-bearing C57 mouse using ^67^Cu-NOTA-PEG_2_Nle-CycMSH_hex_ as an imaging probe at 4 h post-injection. The melanoma lesions (T) are highlighted with an arrow on the image.

^67^Cu-NOTA-PEG_2_Nle-CycMSH_hex_ showed higher tumor/kidney and tumor/liver ratios than ^67^Cu-NOTA-GGNle-CycMSH_hex_. Thus, we further performed SPECT imaging of ^67^Cu-NOTA-PEG_2_Nle-CycMSH_hex_ in B16/F10 melanoma-bearing mice. The representative maximum intensity projection SPECT/CT image of ^67^Cu-NOTA-PEG_2_Nle-CycMSH_hex_ in a B16/F10 melanoma-bearing C57 mouse is presented in Figure 3. In agreement with the biodistribution result, the B16/F10 tumor lesions were clearly imaged at 4 h post-injection using ^67^Cu-NOTA-PEG_2_Nle-CycMSH_hex_ as an imaging probe.

## 4. Discussion

The attractive decay properties and availability of high-quality ^177^Lu are key factors that contribute to the success of ^177^Lu radionuclide therapy [29]. ^67^Cu shares some similar decay properties with ^177^Lu. ^177^Lu is a medium-energy (0.497 MeV) β-emitter with a half-life of 6.7 days, whereas ^67^Cu emits 0.562 MeV β-particles with a half-life of 2.6 days. Meanwhile, the specific activity of ^67^Cu can reach 5.55 GBq/μg (150 mCi/μg) by irradiating an enriched ^68^Zn target via a ^68^Zn(γ,p)^67^Cu reaction [30,31]. From a matched-pair perspective, the identical coordination chemistry and theranostic features of ^67^Cu/^64^Cu make ^67^Cu attractive for potential therapeutic application. Furthermore, both ^177^Lu and ^67^Cu emit imageable γ-photons for SPECT that can be utilized to calculate dosimetry and monitor therapeutic response.

**Figure 4:**
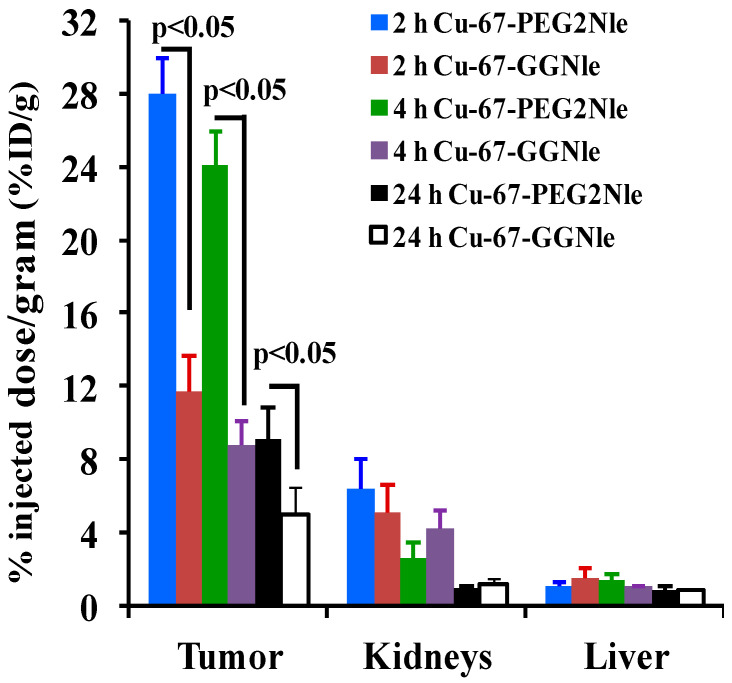
Comparison of uptake in tumor, kidneys, and liver between ^67^Cu-NOTA-PEG_2_Nle-CycMSH_hex_ (PEG_2_Nle) and ^67^Cu-NOTA-GGNle-CycMSH_hex_ (GGNle) at 2, 4, and 24 h post-injection.

We reported the effects of GG, PEG_2_, and Aoc linkers on melanoma uptake of ^64^Cu-NOTA-GGNle-CycMSH_hex_, ^64^Cu-NOTA-PEG_2_Nle-CycMSH_hex_, and ^64^Cu-NOTA-AocNle-CycMSH_hex_ [14,21]. Interestingly, both ^64^Cu-NOTA-GGNle-CycMSH_hex_ and ^64^Cu-NOTA-PEG_2_Nle-CycMSH_hex_ exhibited higher melanoma uptake than ^64^Cu-NOTA-AocNle-CycMSH_hex_ [14,21]. Thus, we further evaluated the tumor targeting and clearance properties of ^67^Cu-NOTA-GGNle-CycMSH_hex_ and ^67^Cu-NOTA-PEG_2_Nle-CycMSH_hex_ in B16/F10 melanoma-bearing C57 mice in this study. First of all, ^67^Cu-NOTA-PEG_2_Nle-CycMSH_hex_ displayed higher MC1R-specific cellular uptake than ^67^Cu-NOTA-GGNle-CycMSH_hex_ on B16/F10 melanoma cells. The cellular uptake of ^64^Cu-NOTA-PEG_2_Nle-CycMSH_hex_ was 2.5 times that of ^64^Cu-NOTA-GGNle-CycMSH_hex_ (Figure 2). Furthermore, ^67^Cu-NOTA-PEG_2_Nle-CycMSH_hex_ exhibited higher MC1R-specific uptake than ^67^Cu-NOTA-GGNle-CycMSH_hex_ on B16/F10 melanoma. The tumor uptake of ^67^Cu-NOTA-PEG_2_Nle-CycMSH_hex_ was 2.4, 2.7, and 1.9 times the tumor uptake of ^67^Cu-NOTA-GGNle-CycMSH_hex_ at 2, 4, and 24 h post-injection, respectively (Figure 4). The B16/F10 melanoma lesions could be clearly visualized using ^67^Cu-NOTA-PEG_2_Nle-CycMSH_hex_ as an imaging probe (Figure 3). It is worthwhile to note that NOTA-PEG_2_Nle-CycMSH_hex_ and NOTA-GGNle-CycMSH_hex_ showed similar nanomolar MC1R binding affinities (1.2 vs. 1.6 nM) [14,21]. Therefore, it is necessary to perform biodistribution studies to fully appreciate the potential difference in tumor uptake of the peptides with similar in vitro binding affinities.

The renal and liver uptake of ^67^Cu-NOTA-PEG_2_Nle-CycMSH_hex_ and ^67^Cu-NOTA-GGNle-CycMSH_hex_ were in a similar range (Figure 4). As compared to ^67^Cu-NOTA-GGNle-CycMSH_hex_, higher tumor uptake of ^67^Cu-NOTA-PEG_2_Nle-CycMSH_hex_ yielded higher tumor/kidney and tumor/liver uptake ratios. As shown in Table 1 and Table 2, the tumor/kidney ratio of ^67^Cu-NOTA-PEG_2_Nle-CycMSH_hex_ was 2.4, 2.7, and 1.9 times the tumor/kidney ratio of ^67^Cu-NOTA-GGNle-CycMSH_hex_ at 2, 4, and 24 h post-injection, respectively. The tumor/liver ratio of ^67^Cu-NOTA-PEG_2_Nle-CycMSH_hex_ was 3.3, 2.0, and 1.8 times the tumor/liver ratio of ^67^Cu-NOTA-GGNle-CycMSH_hex_ at 2, 4, and 24 h post-injection, respectively. The enhanced tumor/kidney and tumor/liver uptake ratios of ^67^Cu-NOTA-PEG_2_Nle-CycMSH_hex_ would facilitate its application in melanoma treatment.

A recent study reported the effectiveness of gastrin-releasing peptide receptor (GRPR)-targeted ^67^Cu-SAR-BBN in PC-3 prostate tumor-bearing mice [26]. Huynh et al. examined the efficacy of the treatment of 6 × 24 MBq of ^67^Cu-SAR-BBN and concluded that the treatment inhibited tumor growth and extended median survival from 34.5 days for the control group to greater than 54 days for the treatment group [26]. Another report directly compared the therapeutic efficacy between somatostatin receptor 2 (SST-2)-targeted ^67^Cu-SarTATE and ^177^Lu-DOTA-TATE in AR42J pancreatic tumor-bearing mice [22]. Specifically, Cullinane et al. examined the effectiveness of a single injection of 5 MBq of ^67^Cu-SarTATE or ^177^Lu-DOTA-TATE and found that the treatment of ^67^Cu-SarTATE and ^177^Lu-DOTA-TATE inhibited tumor growth and extended survival from 12 days in the control group to 21 days in the treatment group [22]. Furthermore, they compared the efficacy of 30 MBq of ^67^Cu-SarTATE or ^177^Lu-DOTA-TATE either as a single administration or as two fractions of even activity 2 weeks apart. The results revealed that the treatment of 2 × 15 MBq of ^67^Cu-SarTATE or ^177^Lu-DOTA-TATE significantly improved the survival over the single dose of 30 MBq by 11 days and 17 days, respectively [22]. These promising preclinical results of ^67^Cu-SarTATE and ^67^Cu-SAR-BBN clearly underscore the suitability and feasibility of receptor-targeted ^67^Cu-peptides for targeted radionuclide therapy.

## 5. Conclusions

The melanoma targeting and biodistribution properties of ^67^Cu-NOTA-PEG_2_Nle-CycMSH_hex_ and ^67^Cu-NOTA-AocNle-CycMSH_hex_ were determined in B16/F10 melanoma-bearing C57 mice. ^67^Cu-NOTA-PEG_2_Nle-CycMSH_hex_ showed higher tumor uptake than ^64^Cu-NOTA-AocNle-CycMSH_hex_ after 2 h post-injection. The favorable tumor targeting and biodistribution properties of ^67^Cu-NOTA-PEG_2_Nle-CycMSH_hex_ underscored its potential as an MC1R-targeted therapeutic peptide for melanoma treatment.

## Figures and Tables

**Table 1:** Biodistribution of ^67^Cu-NOTA-PEG_2_Nle-CycMSH_hex_ in B16/F10 melanoma-bearing C57 mice. The data were presented as percent injected dose/gram or as percent injected dose (Mean ± SD, *n* = 4).

Tissues	0.5 h	2 h	4 h	24 h	2 h NDP Blockade
	Percent injected dose/gram (%ID/g)				
Tumor	8.83 ± 2.19	27.97 ± 1.98	24.10 ± 1.83	9.13 ± 1.66	1.66 ± 0.28 *
Brain	0.04 ± 0.02	0.01 ± 0.01	0.01 ± 0.01	0.04 ± 0.03	0.02 ± 0.01
Blood	0.63 ± 0.26	0.20 ± 0.11	0.05 ± 0.02	0.02 ± 0.01	0.01 ± 0.01
Heart	0.58 ± 0.18	0.79 ± 0.06	0.34 ± 0.08	0.05 ± 0.05	0.11 ± 0.09
Lung	0.76 ± 0.16	0.53 ± 0.19	0.62 ± 0.18	0.35 ± 0.13	0.68 ± 0.29
Liver	0.95 ± 0.19	1.09 ± 0.14	1.41 ± 0.26	0.84 ± 0.15	1.70 ± 0.39
Spleen	0.37 ± 0.10	0.02 ± 0.01	0.01 ± 0.01	0.02 ± 0.01	0.03 ± 0.02
Stomach	1.13 ± 0.36	1.40 ± 0.51	0.77 ± 0.09	0.21 ± 0.09	0.65 ± 0.07
Kidneys	6.43 ± 1.31	6.34 ± 1.63	2.60 ± 0.79	0.90 ± 0.18	4.83 ± 0.72
Muscle	0.48 ± 0.12	0.81 ± 0.12	0.03 ± 0.03	0.01 ± 0.01	0.01 ± 0.01
Pancreas	0.10 ± 0.05	0.47 ± 0.06	0.01 ± 0.01	0.01 ± 0.01	0.08 ± 0.06
Bone	0.60 ± 0.35	0.07 ± 0.04	0.03 ± 0.01	0.03 ± 0.01	0.03 ± 0.01
Skin	1.77 ± 0.28	0.59 ± 0.11	0.18 ± 0.07	0.03 ± 0.02	0.16 ± 0.04
	Percent injected dose (%ID)				
Intestines	0.83 ± 0.09	0.95 ± 0.16	1.40 ± 0.34	0.77 ± 0.12	2.09 ± 0.96
Urine	78.31 ± 3.79	89.08 ± 4.96	91.05 ± 1.20	95.61 ± 0.59	89.34 ± 2.19
	Uptake ratio of tumor/normal tissue				
Tumor/blood	14.02	139.85	482.0	456.50	166.0
Tumor/kidney	1.37	4.41	9.27	10.14	0.34
Tumor/lung	11.62	52.77	38.87	26.09	2.44
Tumor/liver	9.29	25.66	17.09	10.87	0.98
Tumor/muscle	18.39	34.53	803.33	913.0	166.0

* *p* < 0.05 for determining the significance of differences in tumor and kidney uptake between ^67^Cu-NOTA-PEG_2_Nle-CycMSH_hex_ with or without peptide blockade at 2 h post-injection.

**Table 2:** Biodistribution of ^67^Cu-NOTA-GGNle-CycMSH_hex_ in B16/F10 melanoma-bearing C57 mice. The data were presented as percent injected dose/gram or as percent injected dose (Mean ± SD, *n* = 4).

Tissues	0.5 h	2 h	4 h	24 h	2 h NDP Blockade
	Percent injected dose/gram (%ID/g)				
Tumor	16.58 ± 1.40	11.66 ± 1.94	8.79 ± 1.31	4.92 ± 1.58	0.61 ± 0.15 *
Brain	0.07 ± 0.04	0.03 ± 0.02	0.02 ± 0.03	0.02 ± 0.02	0.01 ± 0.01
Blood	1.22 ± 0.08	0.03 ± 0.05	0.06 ± 0.07	0.01 ± 0.01	0.01 ± 0.01
Heart	0.71 ± 0.31	0.04 ± 0.04	0.07 ± 0.06	0.03 ± 0.03	0.04 ± 0.04
Lung	0.54 ± 0.69	0.41 ± 0.20	0.37 ± 0.09	0.27 ± 0.12	0.25 ± 0.16
Liver	1.15 ± 0.07	1.49 ± 0.56	1.02 ± 0.07	0.82 ± 0.12	0.66 ± 0.23
Spleen	0.52 ± 0.32	0.05 ± 0.03	0.12 ± 0.08	0.03 ± 0.01	0.09 ± 0.08
Stomach	1.53 ± 0.53	0.81 ± 0.14	0.71 ± 0.22	0.30 ± 0.17	0.65 ± 0.22
Kidneys	7.18 ± 2.63	5.08 ± 1.52	4.21 ± 0.92	1.19 ± 0.28	3.19 ± 0.89
Muscle	0.51 ± 0.18	0.04 ± 0.04	0.01 ± 0.01	0.01 ± 0.01	0.01 ± 0.01
Pancreas	0.10 ± 0.08	0.12 ± 0.08	0.04 ± 0.04	0.02 ± 0.01	0.02 ± 0.02
Bone	0.68 ± 0.10	0.21 ± 0.24	0.06 ± 0.06	0.13 ± 0.16	0.01 ± 0.01
Skin	2.05 ± 0.46	0.50 ± 0.24	0.27 ± 0.19	0.02 ± 0.01	0.09 ± 0.06
	Percent injected dose (%ID)				
Intestines	1.46 ± 0.49	1.30 ± 0.27	2.70 ± 0.26	0.94 ± 0.32	1.48 ± 0.17
Urine	79.77 ± 2.25	88.97 ± 2.48	86.99 ± 1.22	96.23 ± 1.03	95.14 ± 1.28
	Uptake ratio of tumor/normal tissue				
Tumor/blood	13.59	388.67	146.50	492.0	61.0
Tumor/kidney	2.31	2.29	2.09	4.13	0.19
Tumor/lung	30.70	28.44	23.76	18.22	2.44
Tumor/liver	14.42	7.83	8.62	6.0	0.92
Tumor/muscle	32.51	291.50	879.0	492.0	61.0

* *p* < 0.05 for determining the significance of differences in tumor and kidney uptake between ^67^Cu-NOTA-GGNle-CycMSH_hex_ with or without peptide blockade at 2 h post-injection.

## Data Availability

All data are contained in the manuscript.

## References

[1] Siegel R.L., Miller K.D., Wagle N.S., Jemal A. (2023). Cancer statistics, 2023. CA Cancer J. Clin..

[2] Chapman P.B., Hauschild A., Robert C., Haanen J.B., Ascierto P., Larkin J., Dummer R., Garbe C., Testori A., Maio M. (2011). BRIM-3 Study Group. Improved survival with vemurafenib in melanoma with BRAF V600E mutation. N. Engl. J. Med..

[3] Sosman J.A., Kim K.B., Schuchter L., Gonzalez R., Pavlick A.C., Weber J.S., McArthur G.A., Hutson T.E., Moschos S.J., Flaherty K.T. (2012). Survival in BRAF V600-mutant advanced melanoma treated with vemurafenib. N. Engl. J. Med..

[4] Hodi F.S., O’Day S.J., McDermott D.F., Weber R.W., Sosman J.A., Haanen J.B., Gonzalez R., Robert C., Schadendorf D., Hassel J.C. (2010). Improved survival with ipilimumab in patients with metastatic melanoma. N. Engl. J. Med..

[5] Weber J.S., O’day S., Urba W., Powderly J., Nichol G., Yellin M., Snively J., Hersh E. (2008). Phase I/II study of ipilimumab for patients with metastatic melanoma. J. Clin. Oncol..

[6] Topalian S.L., Sznol M., McDermott D.F., Kluger H.M., Carvajal R.D., Sharfman W.H., Brahmer J.R., Lawrence D.P., Atkins M.B., Powderly J.D. (2014). Survival, durable tumor remission, and long-term safety in patients with advanced melanoma receiving nivolumab. J. Clin. Oncol..

[7] Weiss S.A., Wolchok J.D., Sznol M. (2019). Immunotherapy of melanoma: Facts and hopes. Clin. Cancer Res..

[8] Siegrist W., Solca F., Stutz S., Giuffrè L., Carrel S., Girard J., Eberle A.N. (1989). Characterization of receptors for alpha-melanocyte-stimulating hormone on human melanoma cells. Cancer Res..

[9] Tatro J.B., Wen Z., Entwistle M.L., Atkins M.B., Smith T.J., Reichlin S., Murphy J.R. (1992). Interaction on an α-melanocyte stimulating hormone-diptheria toxin fusion protein with melanotropin receptors in human metastases. Cancer Res..

[10] Yang J., Xu J., Gonzalez R., Lindner T., Kratochwil C., Miao Y. (2018). ^68^Ga-DOTA-GGNle-CycMSH_hex_ targets the melanocortin-1 receptor for melamoma imaging. Sci. Transl. Med..

[11] Guo H., Yang J., Gallazzi F., Miao Y. (2010). Reduction of the ring size of radiolabeled lactam bridge-cyclized alpha-MSH peptide resulting in enhanced melanoma uptake. J. Nucl. Med..

[12] Guo H., Yang J., Gallazzi F., Miao Y. (2011). Effects of the amino acid linkers on melanoma-targeting and pharmacokinetic properties of Indium-111-labeled lactam bridge-cyclized α-MSH peptides. J. Nucl. Med..

[13] Guo H., Gallazzi F., Miao Y. (2012). Ga-67-labeled lactam bridge-cyclized alpha-MSH peptides with enhanced melanoma uptake and reduced renal uptake. Bioconjug. Chem..

[14] Guo H., Miao Y. (2012). Cu-64-labeled lactam bridge-cyclized alpha-MSH peptides for PET imaging of melanoma. Mol. Pharm..

[15] Guo H., Gallazzi F., Miao Y. (2013). Design and evaluation of new Tc-99m-labeled lactam bridge-cyclized alpha-MSH peptides for melanoma imaging. Mol. Pharm..

[16] Guo H., Miao Y. (2014). Introduction of an aminooctanoic acid linker enhances uptake of Tc-99m-labeled lactam bridge-cyclized alpha-MSH peptide in melanoma. J. Nucl. Med..

[17] Guo H., Miao Y. (2013). Melanoma targeting property of a Lu-177-labeled lactam bridge-cyclized alpha-MSH peptide. Bioorg. Med. Chem. Lett..

[18] Yang J., Xu J., Cheuy L., Gonzalez R., Fisher D.R., Miao Y. (2019). Evaluation of a novel Pb-203-labeled lactam-cyclized alpha-melanocyte-stimulating hormone peptide for melanoma targeting. Mol. Pharm..

[19] Xu J., Yang J., Gonzalez R., Fisher D.R., Miao Y. (2019). Melanoma-targeting property of Y-90-labeled lactam-cyclized alpha-melanocyte-stimulating hormone peptide. Cancer Biother. Radiopharm..

[20] Qiao Z., Xu J., Gonzalez R., Miao Y. (2020). Novel [^99m^Tc]-tricarbonyl-NOTA-conjugated lactam-cyclized alpha-MSH peptide with enhanced melanoma uptake and reduced renal uptake. Mol. Pharm..

[21] Qiao Z., Xu J., Gonzalez R., Miao Y. (2022). Novel ^64^Cu-labeled NOTA-conjugated lactam-cyclized alpha-melanocyte-stimulating hormone peptides with enhanced tumor to kidney uptake ratios. Mol. Pharm..

[22] Cullinane C., Jeffery C.M., Roselt P.D., van Dam E.M., Jackson S., Kuan K., Jackson P., Binns D., van Zuylekom J., Harris M.J. (2020). Peptide receptor radionuclide therapy with ^67^Cu-CuSarTATE is highly efficacious against a somatostatin positive neuroendocrine tumor model. J. Nucl. Med..

[23] Keinänen O., Fung K., Brennan J.M., Zia N., Harris M., van Dam E., Biggin C., Hedt A., Stoner J., Donnelly P.S. (2020). Harnessing ^64^Cu/^67^Cu for a theranostic approach to pretargeted radioimmunotherapy. Proc. Natl. Acad. Sci. USA.

[24] Kelly J.M., Ponnala S., Amor-Coarasa A., Zia N.A., Nikolopoulou A., Williams C., Schlyer D.J., DiMagno S.G., Donnelly P.S., Babich J.W. (2020). Preclinical evaluation of a high-affinity sarcophagine-containing PSMA ligand for ^64^Cu/^67^Cu-based theranostics in prostate cancer. Mol. Pharm..

[25] Hao G., Mastren T., Silvers W., Hassan G., Öz O.K., Sun X. (2021). Copper-67 radioimmunotheranostics for simultaneous immunotherapy and immuno-SPECT. Sci. Rep..

[26] Huynh T.T., van Dam E.M., Sreekumar S., Mpoy C., Blyth B.J., Muntz F., Harris M.J., Rogers B.E. (2022). Copper-67-Labeled Bombesin Peptide for Targeted Radionuclide Therapy of Prostate Cancer. Pharmaceuticals.

[27] Dearling J.L.J., van Dam E.M., Harris M.J., Packard A.B. (2021). Detection and therapy of neuroblastoma minimal residual disease using [^64/67^Cu] Cu-SARTATE in a preclinical model of hepatic metastases. EJNMMI Res..

[28] Bailey D.L., Willowson K.P., Harris M., Biggin C., Aslani A., Lengkeek N.A., Stoner J., Eslick M.E., Marquis H., Parker M. (2023). ^64^Cu treatment planning and ^67^Cu therapy with radiolabelled SARTATE ([^64^Cu/^67^Cu]MeCOSAR-Octreotate) in subjects with unresectable multifocal meningioma–initial results for human imaging, safety, biodistribution and radiation dosimetry. J. Nucl. Med..

[29] Dash A., Pillai M.R.A., Knapp F.F. (2015). Production of ^177^Lu for targeted radionuclide therapy: Available options. Nucl. Med. Mol. Imaging.

[30] Ehst D.A., Smith N.A., Bowers D.L., Makarashvili V. (2012). Copper-67 production on electron linacs—Photonuclear technology development. AIP Conf. Proc..

[31] Stoner J., Gardner T., Gardner T. (2016). A comparison of DOTA and DiamSar chelates of high specific activity eLINAC produced ^67^Cu. J. Nucl. Med..

